# Coping strategies to overcome psychological distress and fear during COVID-19 pandemic in Kuwait

**DOI:** 10.1186/s43045-023-00285-6

**Published:** 2023-02-01

**Authors:** Asmaa M. Elaidy, Majeda S. Hammoud, Ahmed N. Albatineh, Fatma Mustafa Ridha, Sabri M. Hammoud, Hala M. Elsadek, Muhammad Aziz Rahman

**Affiliations:** 1 Faculty of Medicine for Girls, Department of Psychiatry, Al-Azhar University, Cairo, Egypt; 2 Kuwait Centre for Mental Health (KCMH), MOH, Kuwait City, Kuwait; 3 Faculty of Medicine, Department of Pediatrics, Kuwait University, Kuwait City, Kuwait; 4 Faculty of Medicine, Department of Community Medicine and Behavioral Sciences, Kuwait University, Kuwait City, Kuwait; 5 Sheikh Jaber Al Ahmed Al Jaber Al Sabah Hospital, MOH, Kuwait City, Kuwait; 6 Faculty of Medicine for Girls, Department of Dermatology and Venereology, Al-Azhar University, Cairo, Egypt; 7 Institute of Health and Wellbeing, Federation University Australia, Berwick, VIC 3806 Australia; 8 Faculty of Public Health, Universitas Airlangga, Surabaya, Indonesia

**Keywords:** Mental health, COVID-19, Corona virus, Psychological distress, Coping, Resilience, Kuwait

## Abstract

**Background:**

The COVID-19 pandemic has detrimental effects on both physical and psychological well-being of community people worldwide. The purpose of this research was to determine coping strategies and the factors associated with psychological distress and fear among adults in Kuwait during the COVID-19 pandemic.

**Results:**

Participants with good-excellent mental health perception had significantly lower prevalence of reporting high psychological distress, while those identified as patients as used health services in the past 4 weeks had significantly higher prevalence of reporting high psychological distress. On the other hand, individuals born in the same country of residence, whose financial situation was impacted by COVID-19 had significantly lower prevalence of reporting high levels of fear from COVID-19. Those with an income source, with co-morbidities, tested negative to COVID-19, being frontline or essential worker, reported medium to high psychological distress and had significantly higher prevalence of high levels of fear of COVID-19.

**Conclusions:**

Mental health services should be provided in addition to the existing services in primary healthcare settings, so that the impact of ongoing pandemic on psychological wellbeing of people in Kuwait can be addressed.

## Background

By mid-2020, the COVID-19 pandemic had reached over 226 million cases and 4.7 million fatalities [1]. The first wave warned of impending disaster; the second wave identified in-country differences in incidence, prevalence, and mortality rates due to COVID-19, as well as identified health system gaps, policy failures; and the third wave exposed additional global social, financial, policy, and management failures in health system management [2]. Several healthcare facilities remained operational during the COVID-19 epidemic, while others offered units for the treatment of COVID-19 patients. It had been suggested that the restricted policies would have had significant short- and long-term implications, including stress-related psychological disorders such as anxiety and depression among the affected individuals [3].

The sudden increase in work at the front lines of treatment, the threat of transmission of the virus to family members, and the subsequent death or illness of a relative or friend as a result of the pandemic had psychological impact on those working in the health sector, particularly healthcare professionals (HCPs) who had direct or indirect contact with COVID-19 [4, 5]. Recent studies have demonstrated that the global increase in the number of cases and deaths, the weakening of many countries’ healthcare systems, and the resulting lack of effective medical treatment all contributed to anxiety, public fear and depression, which drew less attention during the pandemic management [6]. Thus, it is critical to research the psychological well-being of communities during the COVID-19 epidemic. This is especially true in Kuwait, where approximately one-quarter and one-third of the general population, displayed anxiety and depressed symptoms during the epidemic, respectively [7]. Accordingly, the purpose of this research was to determine the factors associated with psychological distress, fear, and coping behaviors among adults in Kuwait during the COVID-19 pandemic.

## Methods

### Study design and settings

This study was part of a global study led by the last author (MAR) [8]. When the COVID-19 outbreak occurred, this cross-sectional study was conducted in Kuwait, which has an area of 17,820 km^2^ and a population of 4,301,539 people (2020 estimate), using web-based online platforms (Facebook, Messenger, Twitter, LinkedIn), text messages and emails. It was carried out between June 2020 and January 2021.

### Study population

Adults aged > 18 years, who resided in Kuwait and were able to reply to an online questionnaire were eligible. As a result, participants in the research comprised members of the general public, healthcare professionals, patients, university students, and university staff. Patients were defined as persons who had seen a general practitioner or an allied health-care facility (for any medical condition, including a COVID-19-related disease) during the preceding 4 weeks. Individuals, who self-identified as being in direct contact with patients or clients throughout the pandemic period, were considered to be frontline or essential service workers.

### Sampling method

To conduct this cross-sectional research, the snowball sampling method, which is a non-probability sampling method, was used. After ensuring that the participants met the eligibility requirements, individuals were invited to participate in the survey. The population of Kuwait was estimated to be 4,270,471 in 2020 [2] — the prevalence of lifetime mental health issues among people in Kuwait was estimated to be 40%— [9], considering 95% confidence intervals and 5% margin of error and to achieve an 80% power, the minimum sample size needed was estimated to be 369 according to OpenEpi software.

### Data collection

Using the Google form, a connection to an online survey questionnaire with an organized framework was produced. Data were collected between November 2020 and January 2021. Information about the study and the permission form were shown on the first screen of the survey form. Participants who provided permission and fulfilled the eligibility requirements, were the only ones who were allowed to go to the following screen which included the questionnaire. The research questionnaire; consisted of 39 items. All of the replies were kept completely confidential. Using various social media platforms, online community networks, staff and student email databases at participating institutions and hospitals, an invitation with a link to the online survey which included the questionnaire and a QR code was sent to all participants. It was also possible to exchange text messages through SMS, Viber, and WhatsApp. Flyers with the study’s QR codes were also distributed and displayed in academic and health-care environments. For the purpose of minimizing selection bias, the survey was open, meaning that anybody who had access to the survey link could take part in it. There were no incentives for taking part in the research.

### Study instruments

Pre-testing of the survey questionnaire was conducted on a variety of electronic devices. Psychological distress was quantified using the 10-item Kessler Psychological Distress Scale (K-10) [10], fear was assessed using the Fear of COVID-19 Scale (FCV-19S), which consists of seven items [11], and coping was assessed using the Brief Resilient Coping Scale (BRCS), a four-item questionnaire [12]. The present research assessed the reliability of such instruments in the English version [13]. The questionnaire including the information on the study and consent forms were translated into Arabic following a standard translation and validation procedure, where it was translated and back-translated. The Arabic questionnaire was pre-tested and finalized with comments from the study team after ethical approval was obtained.

### Data analysis

For data analysis, the statistical software for social sciences (SPSS) version 22 was utilized. Data were examined and cleaned for abnormalities before being recoded appropriately. Counts and percentages were used to represent categorical data, while averages and standard deviations were used to describe continuous variables such as age. If all anticipated cell counts exceeded 5, Pearson chi square of independence was used to investigate associations between categorical variables; otherwise, the Fisher exact test was used. The scale K10 scores were classified as low (scoring 10–15) to moderate to very high (score 16–50), while the BRCS scores were classified as low (score 4–13) to medium to high (score 14-20) resilient copers. Multiple logistic regression was used to estimate the odds ratio (OR) and adjusted OR (AOR) while controlling for possible confounders in order to quantify and assess associations between study outcomes and other factors.

### Ethics

The Human Ethical Committee of Kuwait University’s Health Sciences Center granted approval for this research (VDR/EC/3655), which was conducted in accordance with international standard.

The Human Ethical Committee of Kuwait University’s Health Sciences Centre approved the study (VDR/EC/3655). Data were collected anonymously. Privacy and confidentiality were maintained.

A hotline service with counseling for a psychiatrist was also included to provide stress-relieving activities to any respondent who felt distressed while completing the study questionnaire.

## Results

The final analysis of this research included 415 people. The mean (SD) age was 35.03(10.9) years, with the majority of participants being female (63.1%). The majority (88.7 %) of participants live with family members or children, and 66.3% were born in Kuwait. Around 73.4 % had a bachelor’s degree or above, 24.1 % had ever smoked, and 44.3% had increased their smoking in the previous 4 weeks. Additionally, 71% reported experiencing little to no distress as a result of their shift in job status, and around 41% identified as a frontline or vital service worker. Around 34.3 % of respondents reported providing care to a family member/patient who had a known/suspected case of COVID-19, whereas 64.1% reported having no known exposure to COVID-19. Additionally, 37% identified as patients or utilized health services in the preceding 4 weeks. Around 45.8 % of the study sample reported that COVID-19 had an effect on their financial situation, while 27.0 % revealed having additional comorbidities, such as cardiac disease/stroke, hypertension, hyperlipidemia, diabetes, cancer, or other respiratory illness, and 68.3% reported visiting a healthcare provider in person in the preceding 4 weeks, with the majority (21.4%) consulting a psychiatrist, as shown in Table 1. Finally, almost 68.4 % reported experiencing moderate to severe psychological distress, 16.9 % reported experiencing severe fear of COVID-19, and 84.8 % indicated being low to medium resilient copers, as seen in Tables 2, 3, and 4.

**Table 1:** Characteristics of the study sample in Kuwait (*N* = 415)

Characteristics	*n* (%)
Age (mean)(SD)	35 (10.9)
Range	18 to 72
Age group^a^	
18–29	132 (38.3)
30–59	201 (58.3)
At least 60	12 (3.5)
Gender	
Male	153 (36.9)
Female	262 (63.1)
Living status	
Live without family members (on your own/shared house/others)	47 (11.3)
Live with family members or children	368 (88.7)
Born in the same country of residence	
No	139 (33.7)
Yes	274 (66.3)
Completed level of education	
Grade 1–12	36 (8.7)
Trade/certificate/diploma	74 (17.9)
Bachelor and above	304 (73.4)
Perceived distress due to change of employment status	
Moderate to a great deal	119 (29)
A little to none	291 (71)
Self-identification as a frontline or essential service worker	
Yes	170 (41)
No	245 (59)
COVID-19 impacted financial situation	
Yes	190 (45.8)
No	225 (54.2)
Co-morbidities	
No comorbid conditions	276 (66.5)
Psychiatric/mental health issues	27 (6.5)
Other co-morbidities^b^	112 (27.0)
Smoking	
Ever smoker (daily/non-daily/ex)	100 (24.1)
Never smoker	315 (75.9)
Increased smoking over the last 4 weeks	
No	44 (55.7)
Yes	35 (44.3)
Provided care to a family member/patient with known/suspected case of COVID-19	
Yes	140 (34.3)
No	268 (65.7)
Experience related to COVID-19 pandemic	
No known exposure to COVID-19	262 (64.1)
Tested positive for COVID-19	39 (9.5)
Tested negative for COVID-19 but self-isolating	80 (19.6)
Recent overseas travel history and was in quarantine	28 (6.8)
Self-identification as a patient/use of health service in the last 4 weeks	
No	260 (63)
Yes	153 (37)
Healthcare service use in the last 4 weeks	
Visited healthcare providers in person	97 (68.3)
Telehealth consultation with healthcare providers/national helpline	29 (20.4)
Used both	16 (11.3)
Healthcare service use to overcome COVID-19-related stress in the last 4 weeks	
Yes	42 (10.3)
No	367 (89.7)
Types of mental health service used	
Consulted a GP	8 (19)
Consulted a psychologist	1 (2.4)
Consulted a psychiatrist	9 (21.4)
Used mental health resources	3 (7.1)
Used mental health resources available through media	4 (9.5)
Used mental health support services	3 (7.1)
Used combination of services	14 (33.3)

^a^Some values are missing

^b^Cardiac diseases/stroke/hypertension/hyperlipidaemia/diabetes/cancer/chronic respiratory illness

**Table 2:** Level of psychological distress among study sample in Kuwait (*N* = 415)

Anxiety and Depression Checklist (K10) (last 4 weeks)	*n* (%)
About how often did you feel tired out for no good reason?	
None	102 (24.6)
A little	120 (28.9)
Sometime	120 (28.9)
Most of the time	58 (14.0)
All the time	15 (3.6)
About how often did you feel nervous?	
None	76 (18.3)
A little	131 (31.6)
Sometime	130 (31.3)
Most of the time	46 (11.1)
All the time	32 (7.1)
About how often did you feel so nervous that nothing could calm you down?	
None	186 (44.8)
A little	101 (24.3)
Sometime	87 (21)
Most of the time	26 (6.3)
All the time	15 (3.6)
About how often did you feel hopeless?	
None	210 (50.6)
A little	98 (23.6)
Sometime	67 (16.1)
Most of the time	27 (6.5)
All the time	13 (3.1)
About how often did you feel restless or fidgety?	
None	92 (22.2)
A little	153 (36.9)
Sometime	107 (25.8)
Most of the time	43 (10.4)
All the time	20 (4.8)
About how often did you feel so restless you could not sit still?	
None	211 (50.8)
A little	110 (26.5)
Sometime	56 (13.5)
Most of the time	26 (6.3)
All the time	12 (2.9)
About how often did you feel so depressed?	
None	153 (36.9)
A little	126 (30.4)
Sometime	82 (19.8)
Most of the time	43 (10.4)
All the time	11 (2.7)
About how often did you feel that everything was an effort?	
None	118 (28.4)
A little	110 (26.5)
Sometime	98 (23.6)
Most of the time	53 (12.8)
All the time	36 (8.7)
About how often did you feel so sad that nothing could cheer you up?	
None	159 (38.3)
A little	98 (23.6)
Sometime	97 (23.4)
Most of the time	38 (9.2)
All the time	23 (5.5)
About how often did you feel worthless?	
None	250 (60.2)
A little	69 (16.6)
Sometime	62 (14.9)
Most of the time	20 (4.8)
All the time	14 (3.4)
K10 score	
Mean (±SD)	21.64 (9.2)
Range	10–50
Level of psychological distress (K10 categories)	
Low (score 10–15)	131 (31.6)
Moderate (score 16–21)	110 (26.5)
High (score 22–29)	99 (23.9)
Very high (score 30–50)	75 (18.1)

**Table 3:** Level of fear of COVID-19 among the study participants in Kuwait (*N* = 415)

Fear of COVID-19 Scale (FCV-19S) individual items	n(%)
I am most afraid of COVID-19	
Strongly disagree	114 (27.5)
Somewhat disagree	91 (21.9)
Neither agree nor disagree	100 (24.1)
Somewhat agree	84 (20.2)
Strongly agree	26 (6.3)
It makes me uncomfortable to think about COVID-19	
Strongly disagree	111 (26.7)
Somewhat disagree	78 (18.8)
Neither agree nor disagree	85 (20.5)
Somewhat agree	110 (26.5)
Strongly agree	31 (7.5)
My hands become clammy when I think about COVID-19	
Strongly disagree	273 (65.8)
Somewhat disagree	77 (18.6)
Neither agree nor disagree	43 (10.4)
Somewhat agree	15 (3.6)
Strongly agree	7 (1.7)
I am afraid of losing my life because of COVID-19	
Strongly disagree	170 (41)
Somewhat disagree	77 (18.6)
Neither agree nor disagree	79 (19)
Somewhat agree	55 (13.3)
Strongly agree	34 (8.2)
When watching news and stories about COVID-19 on social media, I become nervous or anxious	
Strongly disagree	133 (32)
Somewhat disagree	65 (15.7)
Neither agree nor disagree	92 (22.2)
Somewhat agree	86 (20.7)
Strongly agree	39 (9.4)
I cannot sleep because I’m worrying about getting COVID-19	
Strongly disagree	278 (67)
Somewhat disagree	73 (17.6)
Neither agree nor disagree	42 (10.1)
Somewhat agree	17 (4.1)
Strongly agree	5 (1.2)
My heart races or palpitates when I think about getting COVID-19	
Strongly disagree	270 (65.1)
Somewhat disagree	72 (17.3)
Neither agree nor disagree	42 (10.1)
Somewhat agree	22 (5.3)
Strongly agree	9 (2.2)
FCV-19S score (total)	
Mean (±SD)	14 (6.43)
Range	7 to 35
Level of fear of COVID-19 (FCV-19S categories)	
Low (score 7–21)	345 (83.1)
High (score 22–35)	70 (16.9)

**Table 4:** Coping during COVID-19 pandemic among the study participants (*N* = 415)

Brief Resilient Coping Scale (BRCS) individual items	n(%)
I look for creative ways to alter difficult situations	
Does not describe me at all	44 (10.6)
Does not describe me	59 (14.2)
Neutral	126 (30.4)
Describes me	140 (33.7)
Describes me very well	46 (11.1)
Regardless of what happens to me, I believe I can control my reaction to it	
Does not describe me at all	24 (5.8)
Does not describe me	42 (10.1)
Neutral	111 (26.7)
Describes me	186 (44.8)
Describes me very well	52 (12.5)
I believe I can grow in positive ways by dealing with difficult situations	
Does not describe me at all	18 (4.3)
Does not describe me	26 (6.3)
Neutral	101 (24.3)
Describes me	210 (50.6)
Describes me very well	60 (14.5)
I actively look for ways to replace the losses I encounter in life	
Does not describe me at all	36 (8.7)
Does not describe me	38 (9.2)
Neutral	127 (30.6)
Describes me	154 (37.1)
Describes me very well	60 (14.5)
BRCS score (total)	
Mean (±SD)	13.73 (3.35)
Range	4 to 20
Level of coping (BRCS categories)	
Low resilient copers (score 4–13)	181 (43.6)
Medium resilient copers (score 14–16)	171 (41.2)
High resilient copers (score 17–20)	63 (15.2)

### Psychological distress

Table 5 summarized the results of the univariate analysis and the OR and AOR. The prevalence of psychological distress was significantly higher among females (OR = 1.199, 95 % CI 1.035, 1.389), those who live with family members (OR = 1.384, 95% CI 1.038, 1.844), those with an income source (OR=1.215, 95% CI 1.060, 1.393), those whose financial situation was impacted by COVID-19 (OR = 1.184, 95% CI 1.040, 1.348), those who reported using health services in the preceding 4 weeks (OR = 1.243, 95%CI 1.098, 1.409), those who reported high level of fear of COVID-19 (OR = 1.405, 95%CI 1.257, 1.570), and those who used healthcare services to alleviate COVID-19-related stress in the preceding 6 months (OR = 1.024, 95%CI 1.026, 1.422).

**Table 5:** Factors associated with high psychological distress among study sample (based on K10 score) (*N*^a^ = 415)

Characteristics	Low (Score 10–15), n(%)	Moderate to Very High (Score 16–50), n(%)	Unadjusted analyses PV (PR: 95% CI)	Adjusted analysis PV (APR: 95% CI)
**Age group**				
18–29 years	30 (22.7)	102 (77.3)	1	1
30–59 years	73 (36.3)	128 (63.7)	**0.007** (0.824: 0.717, 0.947)	**0.008 (0.80: 0.677, 0.945)**
60+ years	7 (58.3)	5 (41.7)	**0.073** (0.539: 0.274, 1.060)	**0.043 (0.492: 0.247, 0.979)**
**Gender**				
Male	60 (39.2)	93 (60.8)	1	1
Female	71 (27.1)	191 (72.9)	**0.015** (1.199: 1.035, 1.389)	0.65 (1.053: 0.843, 1.314)
**Living status**				
Live without family members (on your own/shared house/others)	23 (48.9)	24 (51.1)	1	1
Live with family members (partner and/or children)	108 (29.3)	260 (70.7)	**0.027** (1.384: 1.038, 1.844)	0.158 (1.297: 0.904, 1.86)
**Born in same country of residence**				
No	47 (33.8)	92 (66.2)	1	1
Yes	83 (30.3)	191 (69.7)	0.475 (1.053: 0.914, 1.214)	0.936 (0.992: 0.81, 1.214)
**Completed level of education**				
Grade 1–12	10 (27.8)	26 (72.2)	1	1
Trade/Certificate/Diploma	21 (28.4)	53 (71.6)	0.947 (0.992: 0.774, 1.271)	0.375 (0.864: 0.624, 1.194)
Bachelor and above	100 (32.9)	204 (67.1)	0.508 (0.929: 0.748, 1.155)	0.464 (0.888: 0.646, 1.221)
**Current employment condition**				
Jobs affected by COVID-19 (lost job/working hours reduced/ afraid of job loss)	113 (34.8)	212 (65.2)	1	1
Have an income source (employed/Government benefits)	17 (20.7)	65 (79.3)	**0.005** (1.215: 1.060, 1.393)	0.118 (1.191: 0.957, 1.483)
**Perceived distress due to change of employment status**				
A little to none	97 (33.3)	194 (66.7)	1	1
Moderate to a great deal	33 (27.7)	86 (72.3)	0.251 (1.084: 0.944, 1.244)	0.391 (1.074: 0.912, 1.265)
**Self-identification as a frontline or essential service worker**				
No	75 (30.6)	170 (69.4)	1	1
Yes	56 (32.9)	114 (67.1)	0.618 (0.966: 0.845, 1.105)	0.391 (1.074: 0.912, 1.265)
**COVID-19 impacted financial situation**				
No	83 (36.9)	142 (63.1)	1	1
Yes	48 (25.3)	142 (74.7)	**0.011** (1.184: 1.040, 1.348)	0.616 (1.049: 0.871, 1.262)
**Co-morbidities**^**b**^				
No	98 (35.5)	178 (64.5)	1	1
Yes	33 (23.7)	106 (76.3)	0.010 (1.182: 1.041, 1.343)	0.315 (1.095: 0.917, 1.307)
**Smoking Status**				
Never smoker	98 (31.1)	217 (68.9)	1	1
Ever smoker	33 (33.0)	67 (67.0)	0.727 (0.973: 0.832, 1.14)	0.596 (0.942: 0.754, 1.176)
**Current use of alcohol**				
No	123 (31.1)	272 (68.9)	1	1
Yes	7 (43.8)	9 (56.3)	0.364 (0.817: 0.528, 1.265)	0.529 (0.820: 0.441, 1.524)
**Provided care to a family member/patient with known/suspected case of COVID-19**				
No	86 (32.1)	182 (67.9)	1	1
Yes	43 (30.7)	97 (69.3)	0.775 (1.020: 0.889, 1.171)	0.507 (0.934: 0.764, 1.142)
**Experience related to COVID-19**				
No known exposure to COVID-19	89 (34.0)	173 (66.0)	1	1
Tested positive to COVID-19	9 (23.1)	30 (76.9)	0.120 (1.165: 0.961, 1.412)	0.504 (1.096: 0.838, 1.433)
Tested negative to COVID-19	20 (25.0)	60 (75.0)	0.104 (1.136: 0.974, 1.324)	**0.023 (1.262: 1.033, 1.542)**
Recent overseas travel and was in quarantine	11 (39.3)	67 (60.7)	0.596 (0.919: 0.674, 1.254)	0.329 (0.839: 0.589, 1.194)
**Self-identification as patient/ use of health services use in last 4 weeks**				
No	96 (36.9)	164 (63.1)	1	1
Yes	33 (21.6)	120 (78.4)	**0.001** (1.243: 1.098, 1.409)	**0.004 (1.288: 1.084, 1.530)**
**Mental health perception**				
Poor – fair	4 (3.7)	104 (96.3)	1	1
Good - excellent	127 (41.4)	180 (58.6)	**0.001** (0.609: 0.550, 0.674)	**0.001 (0.68: 0.597, 0.775)**
**Level of fear of COVID-19 (FCV-19S categories)**				
Low (score 7–21)	124 (35.9)	221 (64.1)	1	1
High (score 22–35)	7 (10.0)	63 (90)	**0.001** (1.405: 1.257, 1.570)	**0.025 (1.22: 1.025, 1.453)**
**Level of coping (BRCS categories)**				
Low resilient (score 4–13)	44 (24.3)	137 (75.7)	1	1
Medium - high resilient (score 14–20)	87 (37.2)	147 (62.8)	**0.004** (0.830: 0.730, 0.944)	0.458 (0.940: 0.798, 1.107)
**Health care service use to overcome COVID-19 related stress in last 6 months**				
No	121 (33.0)	246 (67.0)	1	1
Yes	8 (19.0)	34 (81.0)	**0.024** (1.208: 1.026, 1.422)	0.632 (0.948: 0.764, 1.178)

*PV* probability value, *PR* prevalence ratio, *APR* adjusted prevalence ratio

^a^Some values are missing

^b^Cardiac diseases/stroke/hypertension/hyperlipidaemia/diabetes/cancer/chronic respiratory illness

However, after adjusting for potential confounders, the adjusted model revealed that participants who tested negative (AOR = 1.262, 95%CI 1.033, 1.542), those who identified themselves as using health services in the last 4 weeks (AOR = 1.288, 95%CI 1.084, 1.530), and those who reported high level of fear of COVID-19 (AOR = 1.22, 95%CI 1.025, 1.453) had significantly higher prevalence of psychological distress. In contrast, older individuals (AOR = 0.492, 95% CI 0.247, 0.979), as well as those who reported good to outstanding mental health (AOR = 0.68, 95% CI 0.597, 0.775), had a considerably lower prevalence of psychological distress, as shown in Table 5.

### Levels of fear

Table 6 showed the univariate OR and AOR distress with 95% CI were reported in Table 6. Univariate analysis revealed that the prevalence of fear of COVID-19 was significantly higher among females (APR = 1.821, 95%CI 1.095, 3.028), in the age group 30–59 years (APR = 1.777, 95%CI 1.066, 2.963), those with co-morbidities (APR = 2.807, 95%CI 1.827, 4.314), those who tested negative to COVID-19 (APR = 2.283, 95%CI 1.427, 3.652), identified themselves as patients/used health services in the last 4 weeks (APR = 1.799, 95%CI 1.177, 2.750), scored medium to very high score on psychological distress (K10) (APR = 4.151, 95%CI 1.955, 8.814), and those who used health care services to overcome COVID-19-related stress in the last 6 months (APR = 2.065, 95%CI 1.236, 3.451), while those who identified themselves as frontline or essential worker showed marginal significance (*P* value = 0.092, APR = 1.441, 95%CI 0.942, 2.206). On the other hand, the prevalence of fear of COVID-19 was significantly lower among individuals born in the same nation (APR = 0.479, 95%CI 0.314, 0.731).

**Table 6:** Factors associated with levels of fear of COVID-19 among study sample (based on FCV-19S score) (*N*^a^ = 415)

Characteristics	Low (score 7–21), *n*(%)	High (score 22–35), *n*(%)	Unadjusted analyses PV (PR: 95% CI)	Adjusted analysis PV (APR: 95% CI)
**Age group**				
18–29 years	115 (87.1)	17 (12.9)	1	1
30–59 years	155 (77.1)	46 (22.9)	0.027 (1.777: 1.066, 2.963)	0.177 (1.428: 0.852, 2.396)
60+ years	11 (91.7)	1 (8.3)	0.658 (0.647: 0.094, 4.450)	0.397 (0.381: 0.041, 3.562)
**Gender**				
Male	136 (88.9)	17 (11.1)	1	1
Female	209 (79.8)	53 (20.2)	0.021 (1.821: 1.095, 3.028)	0.346 (1.430: 0.680, 3.009)
**Living status**				
Live without family members (on your own/shared house/others)	41 (87.2)	6 (12.8)	1	1
Live with family members (partner and/or children)	304 (82.6)	64 (17.4)	0.437 (1.362: 0.625, 2.971)	0.339 (1.70: 0.573, 5.046)
**Born in same country of residence**				
No	103 (74.1)	36 (25.9)	1	1
Yes	240 (87.6)	34 (12.4)	0.001 (0.479: 0.314, 0.731)	**0.025 (0.547: 0.323, 0.926)**
**Completed level of education**				
Grade 1–12	32 (88.9)	4 (11.1)	1	1
Trade/certificate/diploma	64 (86.5)	10 (13.5)	0.725 (1.216: 0.409, 3.614)	0.951 (1.033: 0.374, 2.851)
Bachelor and above	249 (81.9)	55 (18.1)	0.317 (1.628: 0.627, 4.229)	0.628 (1.238: 0.522, 2.935)
**Current employment condition**				
Jobs affected by COVID-19 (lost job/working hours reduced/ afraid of job loss)	272 (83.7)	53 (16.3)	1	1
Have an income source (employed/government benefits)	66 (80.5)	16 (19.5)	0.485 (1.197: 0.723, 1.980)	**0.012 (2.441: 1.218, 4.893)**
**Perceived distress due to change of employment status**				
A little to none	242 (83.2)	49 (16.8)	1	1
Moderate to a great deal	99 (83.2)	20 (16.8)	0.994 (0.998: 0.621, 1.604)	0.598 (0.869: 0.516, 1.464)
**Self-identification as a frontline or essential service worker**				
No	210 (85.7)	35 (14.3)	1	1
Yes	135 (79.4)	35 (20.6)	0.092 (1.441: 0.942, 2.206)	**0.065 (1.663: 0.969, 2.855)**
**COVID-19 impacted financial situation**				
No	186 (82.7)	39 (17.3)	1	1
Yes	159 (83.7)	31 (16.3)	0.783 (0.941: 0.612, 1.447)	**0.020 (0.566: 0.350, 0.915)**
**Co-morbidities**^**b**^				
No	247 (89.5)	29 (10.5)	1	1
Yes	98 (70.5)	41 (29.5)	0.001 (2.807: 1.827, 4.314)	**0.001 (2.918: 1.721, 4.948)**
**Smoking Status**				
Never smoker	260 (82.5)	55 (17.5)	1	1
Ever smoker	85 (85.0)	15 (15.0)	0.571 (0.859: 0.508, 1.452)	0.678 (0.856: 0.412, 1.782)
**Current use of alcohol (last 4 weeks)**				
No	327 (82.8)	68 (17.2)	1	1
Yes	14 (87.5)	2 (12.5)	0.633 (0.726: 0.195, 2.703)	0.951 (0.966: 0.323, 2.892)
**Provided care to a family member/patient with known/suspected case of COVID-19**				
No	229 (85.4)	39 (14.6)	1	1
Yes	112 (80.0)	28 (20.0)	0.157 (1.374: 0.885, 2.135)	0.528 (1.217: 0.661, 2.242)
**Experience related to COVID-19**				
No known exposure to COVID-19	229 (87.4)	33 (12.6)	1	1
Tested positive to COVID-19	32 (82.1)	7 (17.9)	0.350 (1.425: 0.678, 2.996)	0.470 (1.435: 0.539, 3.821)
Tested negative to COVID-19	57 (71.3)	23 (28.7)	0.001 (2.283: 1.427, 3.652)	**0.040 (1.848: 1.028, 3.32)**
Recent overseas travel and was in quarantine	22 (78.6)	6 (21.4)	0.180 (1.701: 0.782, 3.703)	0.564 (1.370: 0.470, 3.997)
**Self-identification as patient/use of health services use in last 4 weeks**				
No	226 (86.9)	34 (13.1)	1	1
Yes	117 (76.5)	36 (23.5)	0.007 (1.799: 1.177, 2.750)	0.737 (1.103: 0.622, 1.955)
**Mental health perception**				
Poor–fair	78 (72.2)	30 (27.8)	1	1
Good−excellent	267 (87.0)	40 (13.0)	0.001 (0.469: 0.308, 0.714)	0.202 (0.719: 0.432, 1.194)
**Level psychological distress (K10 categories)**				
Low (score 10−15)	124 (94.7)	7 (5.3)	1	1
Medium to very high (score 16–50)	221 (77.8)	63 (22.2)	0.001 (4.151: 1.955, 8.814)	**0.027 (2.60: 1.117, 6.049)**
**Level of coping (BRCS categories)**				
Low resilient (score 4–13)	147 (81.2)	34 (18.8)	1	1
Medium−high resilient (score 14–20)	198 (84.6)	36 (15.4)	0.359 (0.819: 0.535, 1.255)	0.692 (1.10: 0.687, 1.762)
**Health care service use to overcome COVID-19-related stress in last 6 months**				
No	312 (85.0)	55 (15.0)	1	1
Yes	29 (69.0)	13 (31.0)	0.006 (2.065: 1.236, 3.451)	0.14 (1.634: 0.851,3.139)

*PV* probability value, *PR* prevalence ratio, *APR* adjusted prevalence ratio

^a^Some values are missing

^b^Cardiac diseases/stroke/hypertension/hyperlipidaemia/diabetes/cancer/chronic respiratory illness

However, after adjusting for potential confounders, the prevalence of fear of COVID-19 was significantly higher among those an income source/government benefits (APR = 2.441, 95%CI 1.218, 4.893), with co-morbidities (APR = 2.918, 95%CI 1.721, 4.948), tested negative for COVID-19 (APR = 1.848, 95%CI 1.028, 3.320), scored medium to very high on the psychological distress scale (K10), (APR = 2.60, 95%CI 1.117, 6.049), while those self-identified as frontline or essential worker had marginally significantly higher level of fear of COVID-19 (*P* value = 0.065, APR = 1.663, 95%CI 0.969, 2.855). On the other hand, the prevalence of fear of COVID-19 was significantly lower among those born in the same country of residence (APR = 0.547, 95%CI 0.323, 0.926), those for which COVID-19 impacted their financial situation (APR = 0.566, 95%CI 0.350, 0.915), see Table 6.

### Coping strategies

In the adjusted model, in order to compare medium to high resilient copers against low resilience copers, the results indicated that the prevalence of medium to high resilience was significantly higher among those who reported good to excellent mental health (APR=1.795, 95%CI 1.271, 2.535) depending on the BRCS scale score. On the other hand, those who were born in the same country of residence (APR = 0.766, 95%CI 0.610, 0.926) and those with moderate to a great deal of perceived distress due to change of employment status had significantly lower prevalence of medium to high resilience (APR = 0.578, 95%CI 0.436, 0.766). All other covariates were not significant at the 5% level.

## Discussion

This cross-sectional research was one of the first studies conducted among people of Kuwait with an objective of investigating the extent of and identify variables linked with psychological distress, levels of fear, and coping methods during the COVID-19 pandemic.

The present research found that almost 68.4% expressed moderate to high psychological distress, and 16.9% reported high level of fear of COVID-19, whereas about 84.8% described being low to medium resilient coppers.

We also compared median scores for psychological distress, fear of COVID-19, and resilience coping among people who had contact with known/suspected COVID-19 patients. Results indicated that people who had contact with known/suspected COVID-19 patients had higher median psychological distress scores and higher median fear of COVID-19 scores but not significantly higher. Furthermore, we have compared the median score for psychological distress, fear of COVID-19, and resilience coping among healthcare workers (doctors, nurses, etc.) and others. Results indicated that the distribution of resilience coping scores was significantly different (*p* value = 0.023) between healthcare workers and others. Also, results indicated that healthcare workers had marginally significantly higher (*p* value = 0.060) median fear of COVID-19 scores compared to non-healthcare workers.

During the COVID-19 pandemic in Australia, Rahman et al. [8] conducted a cross sectional online survey among Australian residents, including patients, frontline health and other essential service workers, and community members during June 2020 to investigate factors associated with psychological distress, fear and coping strategies during the COVID-19 pandemic in Australia and reported comparable results that more than two-thirds (69%) participants experienced moderate to very high levels of psychological distress and about a quarter (24%) had a high level of fear of COVID-19 and more than half of the participants (57%) reported medium to high levels of resilient coping.

Moreover, the study’s findings suggested that the low levels of anxiety among frontline health practitioners were likely to be related to their prolonged personal exposure to COVID-19 patient therapy [1].

Additionally, earlier studies showed that almost a third of individuals (33%) had moderate to severe psychological distress; nevertheless, they discovered that a greater proportion of people exhibited a high degree of anxiety of COVID-19 (32%). Additionally, the Australian research discovered that almost all participants (97%) had low resilient coping. Learning from prior successful experiences that help individuals to cope more effectively may account for this disparity [14].

The acquired statistics in current study are consistent with Rahman et al. [8] who evaluated the amount and variables related with psychological distress, fear of COVID-19, and coping. Their findings indicated that 62.6% of research participants had moderate to severe psychological distress.

Our findings corroborate Wang et al. [15], who reported that a recent survey conducted in 194 cities throughout China found that 53.8% rated psychological damage as moderate to severe.

According to the current research, the incidence of psychological distress was considerably greater among individuals who identified as having used health services in the previous 4 weeks and those who expressed a high degree of dread of COVID-19. On the other hand, older adults with good to excellent mental health reported a much reduced incidence of psychological distress.

In accordance with our results, Rahman et al. [1] reported that higher psychological distress was associated with pre-existing mental health conditions, increased smoking in the last 4 weeks and higher levels of fear of COVID-19 while lower psychological distress was associated with being older (60+ years) and being a frontline or essential service worker.

Aon et al. [16] reported comparable results in a cross-sectional survey of health care workers (HCWs) during the COVID-19 pandemic in Kuwait. The findings indicated that medical staff suffers from psychological distress, including anxiety and stress, as a result of a variety of factors, including exposure to COVID-19 at work and fear of spreading the infection to their family, the increasing flow of COVID-19 cases, fear of not having rapid access to testing if they develop COVID-19 symptoms, long work hours, and physical exhaustion.

The findings corroborate those of Aon et al. [16] who concluded that younger age, medical employment, and female gender are all risk factors for moderate psychological distress. While being a physician and being younger in age are risk factors for severe psychological distress.

Additionally, young HCWs with junior healthcare professionals suffered psychological stress as a result of their increased interaction with patients and potential exposure to moral harm as a result of patients’ suffering and death [17].

Collectively, psychological distress among HCWs were associated with higher interaction with infected patients, extended isolation, felt societal stigma directed against HCWs, a previous history of psychiatric diseases, and a reported lack of organizational support [17].

Additionally, the current study’s findings indicated that fear of COVID-19 was significantly higher among those with an income source/government benefits, those with co-morbidities, those who tested negative for COVID-19, and those who scored medium to very high on the psychological distress scale (K10), whereas those who self-identified as frontline or essential workers had a marginally higher level of fear of COVID-19. On the other hand, fear of COVID-19 was much lower among individuals born in the same country of residency and those whose financial condition was impacted by COVID-19.

Our results were in harmony with Mistry et al. [18] who examined fear of COVID-19 and its associations in Bangladeshi older persons. The study’s results reveal that the COVID-19 epidemic instilled great fear in the elderly. Additionally, the current research identified characteristics associated with COVID-19-related fear, revealing that older persons who were presently unemployed or retired were considerably more likely to be fearful of COVID-19. Participants who had financial troubles during the pandemic expressed greater fear of it. Additionally, those who were worried about the COVID-19 influence and were overwhelmed by it expressed greater fear than those who were indifferent to it. Additionally, psychological stress is thought to be a trigger for COVID-19 fear. The elderly who had difficulty accessing medication and those who suffer from various comorbidities and, in instances with multiple comorbidities were more fearful during this pandemic. Notably, fear levels were considerably lower among those who got information about COVID-19 from health care personnel.

Our results were in the line with Aon et al. [16] who found that during the COVID-19 pandemic in Kuwait, perceived fear of infection was high among 39% of HCWs, while fear about infection transfer to family members was high among 65% of responder medical professionals. The study’s majority of HCWs (80%) realize their anxiety and depression are connected to the COVID-19 pandemic.

In addition, Fares et al. [19] reported comparable results in their data to generate a psychometrically satisfactory instrument to evaluate psychological distress during the COVID-19 disease outbreak across Arab countries. The findings indicated that there was a common physiological sensation related with clinically increased fear and anxiety as a consequence of so much concern regarding coronavirus infection.

Similar findings were also described in Greece during the H1N1 pandemic, when HCWs’ major fear was infecting family and friends (54.9%) [20].

Providing proper Personal protection equipment (PPE) and early examination and testing in the occurrence of symptoms reduces the risk of HCWs acquiring the virus or acting as a pathway for transmission to family members [21].

According to the findings of the present study, using the BRCS scale score and comparing medium to high resilient copers to low resilient copers in an adjusted model revealed that the prevalence of medium to high resilience was significantly higher among those who reported having good to excellent mental health. On the other hand, individuals born in the same country of residence and those experiencing moderate to severe distress as a result of changing work status had a much lower incidence of medium to high resilience.

The present outcomes are in agreement with Rahman et al. [22] who showed that despite experiencing moderate to severe psychological distress and anxiety, more than half of participants (57%) reported having a moderate to high degree of resilient coping. Additionally, Friesen et al. [23] discovered that weaker resilient coping is connected with increased stress levels.

Furthermore, Van Hoof et al. [24] demonstrated that the elderly were typically more resilient than other age groups, despite their increased susceptibility to COVID-19. Additionally, their resilience shielded them against mental health concerns. That might be because elderly people were more self-confident and had accumulated more life experience. In general, it appears as if the coping methods that were present and employed at the time of a crisis had a significant effect on one’s degree of resilience. The elderly might already know which coping strategies will be most beneficial for them as a result of their life experiences, but other age groups must develop these skills. Additionally, Van Hoof et al. [24] showed that mental health had a broad influence on coping.

In families of autistic persons, resilient coping had been connected with self-efficacy, coping style, social support, cognitive evaluation, locus of control, optimism, acceptance, life satisfaction, and positive family functioning [25].

### Study limitations

This research had few limitations. Due to the use of an online self-administered questionnaire, response and memory bias could have been introduced. Additionally, the recruiting process included the distribution of questionnaires through social media platforms, which indicated restriction of participants who had access to and literate to use of internet.

## Conclusions

This research identified those at high risk of psychological distress or fear during Kuwait’s COVID-19 outbreak. Mental health support should be incorporated with current service delivery in primary healthcare settings to help the community people in Kuwait during crisis periods like the current ongoing COVID-19 pandemic.

## Data Availability

All data generated or analyzed during this study are included in this published article.

## Abbreviations

COVID-19: Coronavirus disease 2019
FCV-19S: Fear of COVID-19 Scale
k-10: Kessler Psychological Distress Scale
BRCS: Brief Resilient Coping Scale
HCWs: Health care workers

## References

[1] Rahman MA, Hoque N, Alif SM, Salehin M, Islam SMS, Banik B (2020). Factors associated with psychological distress, fear and coping strategies during the COVID-19 pandemic in Australia. Glob Health.

[2] Alsairafi Z, Naser A, Alsaleh FM, Awad A, Jalal Z (2021). Mental health status of healthcare professionals and students of health sciences faculties in Kuwait during the COVID-19 pandemic. Int J Environ Res Public Health.

[3] Wang C, Tee M, Roy AE, Fardin MA, Srichokchatchawan W, Habib HA (2021). The impact of COVID-19 pandemic on physical and mental health of Asians: a study of seven middle-income countries in Asia. PLoS One.

[4] Maunder R, Hunter J, Vincent L, Bennett J, Peladeau N, Leszcz M (2003). The immediate psychological and occupational impact of the 2003 SARS outbreak in a teaching hospital. CMAJ.

[5] Greenberg N, Docherty M, Gnanapragasam S, Wessely S (2020). Managing mental health challenges faced by healthcare workers during covid-19 pandemic. BMJ.

[6] Chen Q, Liang M, Li Y, Guo J, Fei D, Wang L (2020). Mental health care for medical staff in China during the COVID-19 outbreak. Lancet Psychiatry.

[7] Burhamah W, AlKhayyat A, Oroszlányová M, AlKenane A, Almansouri A, Behbehani M (2020). The psychological burden of the COVID-19 pandemic and associated lockdown measures: experience from 4000 participants. J Affect Disord.

[8] Rahman MA, Islam SMS, Tungpunkom P (2021). COVID-19: factors associated with psychological distress, fear, and coping strategies among community members across 17 countries. Glob Health.

[9] Kaladchibachi S, Al-Dhafiri AM (2018). Mental health care in Kuwait: toward a community-based decentralized approach. Int Soc Work.

[10] Furukawa TA, Kessler RC, Slade T, Andrews G (2003). The performance of the K6 and K10 screening scales for psychological distress in the Australian National Survey of Mental Health and Well-Being. Psychol Med.

[11] Malik S, Ullah I, Irfan M, Ahorsu DK, Lin C-Y, Pakpour AH (2021). Fear of COVID-19 and workplace phobia among Pakistani doctors: a survey study. BMC Public Health.

[12] Sinclair VG, Wallston KA (2004). The development and psychometric evaluation of the Brief Resilient Coping Scale. Assessment.

[13] Rahman MA, Salehin M, Islam SMS, Alif SM, Sultana F, Sharif A (2021). Reliability of the tools used to examine psychological distress, fear of COVID-19 and coping amongst migrants and non-migrants in Australia. Int J Ment Health Nurs.

[14] Chen L, Qu L (2021). From stressful experiences to depression in Chinese migrant children: the roles of stress mindset and coping. Front Psychol.

[15] Wang C, Pan R, Wan X, Tan Y, Xu L, Ho CS (2020). Immediate psychological responses and associated factors during the initial stage of the 2019 coronavirus disease (COVID-19) epidemic among the general population in China. Int J Environ Res Public Health.

[16] Aon MH, Al-Shammari OZ, Aljenfawi MK, Aoun AH (2020). Psychological impact of the covid-19 pandemic on healthcare workers in Kuwait. Am J Res Med Sci.

[17] Stroe AZ, Stuparu AF, Axelerad SD, Axelerad DD, Moraru A (2021). Neuropsychological symptoms related to the COVID-19 pandemic experienced by the general population and particularly by the healthcare personnel. J Mind Med Sci.

[18] Mistry SK, Ali ARMM, Akther F, Yadav UN, Harris MF (2021). Exploring fear of COVID-19 and its correlates among older adults in Bangladesh. Glob Health.

[19] Fares ZEA, Ala’a B, Gadelrab HF, Lin C-Y, Aljaberi MA, Alhuwailah A (2021). Arabic COVID-19 Psychological Distress Scale: development and initial validation. BMJ Open.

[20] Goulia P, Mantas C, Dimitroula D, Mantis D, Hyphantis T (2010). General hospital staff worries, perceived sufficiency of information and associated psychological distress during the A/H1N1 influenza pandemic. BMC Infect Dis.

[21] Shanafelt T, Ripp J, Trockel M (2020). Understanding and addressing sources of anxiety among health care professionals during the COVID-19 pandemic. JAMA.

[22] Rahman MA, Islam SMS, Tungpunkom P, Sultana F, Alif SM, Banik B (2021). COVID-19: Factors associated with psychological distress, fear, and coping strategies among community members across 17 countries. Glob Health.

[23] Friesen KA, Weiss JA, Howe SJ, Kerns CM, McMorris CA (2021) Mental Health and Resilient Coping in Caregivers of Autistic Individuals during the COVID-19 Pandemic: Findings from the Families Facing COVID Study. J Autism Dev Disord 8:1–11 10.1007/s10803-021-05177-4 PMC8265288 34240291

[24] Van Hoof E, De Laet H, Hochrath S, Philips E, Van den Cruyce N (2021). Unravelling the impact of COVID-19 on mental health: a scoping review on traumatogenic events using the phases of response to disaster model. Arch Community Med Public Health.

[25] Iacob CI, Avram E, Cojocaru D, Podina IR (2020). Resilience in familial caregivers of children with developmental disabilities: a meta-analysis. J Autism Dev Disord.

